# Suicide‐specific mortality among patients with treatment‐resistant major depressive disorder, major depressive disorder with prior suicidal ideation or suicide attempts, or major depressive disorder alone

**DOI:** 10.1002/brb3.3171

**Published:** 2023-07-21

**Authors:** David M. Kern, Carla M. Canuso, Ella Daly, Jonathan C. Johnson, Dong Jing Fu, Teodora Doherty, Cori Blauer‐Peterson, M. Soledad Cepeda

**Affiliations:** ^1^ Department of Epidemiology Janssen Research & Development Titusville New Jersey United States; ^2^ Optum Eden Prairie Minnesota

**Keywords:** attempted suicide, major depressive disorder, mortality, suicidal ideation, suicide, treatment‐resistant depression

## Abstract

**Background:**

The impact of treatment‐resistant depression (TRD) or prior suicidal ideation/suicide attempt (SI/SA) on mortality by suicide among patients with major depressive disorder (MDD) is not well known. This retrospective, observational, descriptive cohort study characterized real‐world rates of suicide‐specific mortality among patients with MDD with or without TRD or SI/SA.

**Methods:**

Adult patients with MDD among commercially insured and Medicare enrollees in Optum Research Database were included and assigned to three cohorts: those with treatment‐resistant MDD (TRD), those with MDD and SI/SA (MDD+SI/SA), and those with MDD without TRD or SI/SA (MDD alone). Suicide‐specific mortality was obtained from the National Death Index. The effects of demographic characteristics and SI/SA in the year prior to the end of observation on suicide‐specific mortality were assessed.

**Results:**

For the 139,753 TRD, 85,602 MDD+SI/SA, and 572,098 MDD alone cohort patients, mean age ranged from 55 to 59 years and the majority were female. At baseline, anxiety disorders were present in 53.92%, 44.11%, and 21.72% of patients with TRD, MDD+SI/SA, and MDD alone, respectively. Suicide‐mortality rates in the three cohorts were 0.14/100 person‐years for TRD, 0.27/100 person‐years for MDD+SI/SA, and 0.04/100 person‐years for MDD alone. SI/SA during the year prior to the end of observation, younger age, and male sex were associated with increased suicide risk.

**Conclusions:**

Patients with TRD and MDD+SI/SA have a heightened risk of mortality by suicide compared with patients with MDD alone. Suicide rates were higher in patients with recent history versus older or no history of SI/SA, men versus women, and those of young age versus older age.

## Graphical abstract

This study examined nearly 800,000 patients with treatment resistant depression (TRD), major depressive disorder (MDD) with suicidal ideation/suicide attempt (MDD+SI/SA) and those with MDD without SI/SA or TRD (MDD alone). Suicide rates were 0.14/100 person‐years, 0.27/100 person‐years, and 0.04/100 person‐years in the TRD, MDD+SI/SA, and MDD alone cohorts, respectively. SI/SA during the last year of observation, younger age, and male sex were associated with increased suicide risk.

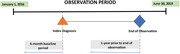

## 1 INTRODUCTION

As one of the leading causes of disability, major depressive disorder (MDD) has been ranked as the third greatest cause of disease burden worldwide and is projected to rank first within the next decade (Bains & Abdijadid, 2021; Malhi & Mann, 2018; WHO, 2017). In the United States, the 12‐month prevalence of MDD is 10.4%, and the lifetime prevalence is 20.6% (Hasin et al., 2018; WHO, 2017). MDD is often a chronic, recurrent illness, with a recurrence rate of approximately 50% after the first, 70% after the second, and 90% after the third depressive episode (Bains & Abdijadid, 2021).

While many treatment modalities exist, 10%−30% of patients with MDD do not respond to oral antidepressant medications (Al‐Harbi, 2012; Kaur & Sanches, 2021; Khan & Brown, 2015; Cepeda et al., 2018; Zhdanava et al., 2021). Patients with MDD who fail to achieve adequate response to two or more antidepressant treatments within the current depressive episode are considered to have treatment‐resistant depression (TRD) (Kaur & Sanches, 2021; Kubitz et al., 2013; McIntyre et al., 2014). Results from the Sequenced Treatment Alternatives to Relieve Depression (STAR*D) study, which was conducted to guide selection of subsequent treatments for patients with MDD who did not respond to or tolerate treatment, showed that patients requiring additional treatment steps (i.e., predefined treatment transitions following treatment intolerance or lack of remission) had increasingly lower remission rates (remission rates of 36.8%, 30.6%, 13.7%, and 13.0% for the first, second, third, and fourth treatment steps, respectively) and higher relapse rates, highlighting the challenges in treating patients with TRD (DiBernardo et al., 2018; Rush et al., 2006).

Compared with patients with MDD alone, patients with TRD have an increased frequency of substance use disorders, anxiety, insomnia, pain, and other psychiatric conditions (Cepeda et al., 2018). Furthermore, patients with TRD appear to have more pronounced decreases in daily functioning and health‐related quality of life (Cepeda et al., 2018; DiBernardo et al., 2018; Johnston et al., 2019). In addition, patients with TRD experience a more severe and protracted course of illness, have significant short‐ and long‐term social impairment, and are more likely to attempt suicide compared with patients with treatment‐responsive MDD (Li & Zheng et al., 2019; Madsen et al., 2021; Vergunst et al., 2013).

Globally, more than 60% of individuals who have attempted suicide have MDD, and patients with this disorder have a 20‐fold higher risk of suicide than the general population (Borentain et al., 2020; Nock et al., 2009; Xin et al., 2018). The prevalence of suicidality, including suicidal ideation (SI) and suicide attempts (SA), is increased in patients with MDD compared with matched controls without MDD (Cai et al., 2021; Center for Behavioral Health Statistics & Quality, 2018). Among people who die by suicide, MDD is the most prevalent mental health diagnosis recorded (Bertolote et al., 2002; Brådvik, 2018; Yeh et al., 2019). In a comparative analysis of patients with TRD and managed depression, which used Medicare Standard Analytic File claims for a nationally representative 5% sample of fee‐for‐service beneficiaries from 2001 to 2009, the SA or self‐inflicted injury rate was sevenfold higher in patients with TRD compared with those with treatment‐responsive MDD (Feldman et al., 2013).

While all‐cause mortality and suicidality among patients with MDD have been well researched, there is a paucity of data assessing rates of suicidality in patients with MDD that is treatment resistant or in patients with MDD who have a history of suicidal behavior. The current study was conducted to assess real‐world rates of suicide‐specific mortality among cohorts of adult patients with MDD that was treatment‐resistant (TRD cohort), patients with MDD and a history of SI/SA (MDD+SI/SA cohort), and patients with MDD without SI/SA or TRD (MDD alone cohort).

## 2 METHODS

### 2.1 Study design

**FIGURE 1:**
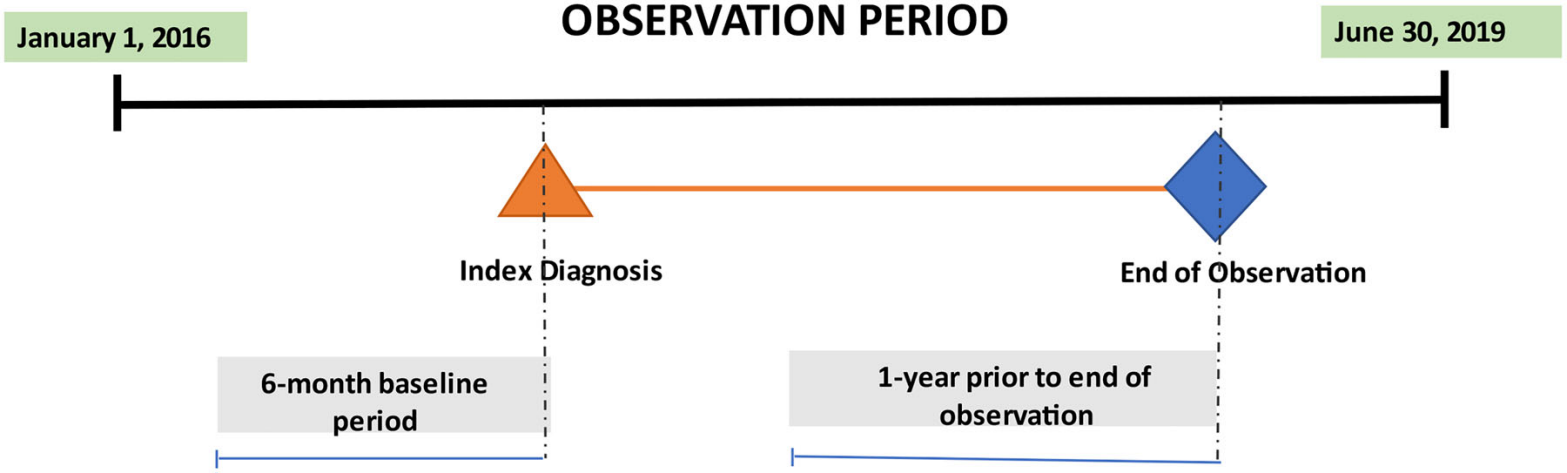
**Study design**. This study was designed to compare rates of suicide‐specific mortality in adults with evidence of ≥2 medical claims (diagnosis of MDD) on separate dates during the observation period (January 1, 2016 and extending through the end of observation [earliest of a patient's health plan disenrollment, death, or June 30, 2019]). The sampling frame included patients who received a diagnosis of TRD, MDD+SI/SA, or MDD alone who had at least a 6‐month history prior to the index diagnosis date (earliest date of service associated with a qualifying claim for TRD, MDD+SI/SA, or MDD alone). Presence of recent SI/SA was captured in the year prior to end of observation. MDD, major depressive disorder; SA, suicidal attempt; SI, suicidal ideation; TRD, treatment‐resistant depression.

This retrospective, observational, descriptive cohort study was designed to estimate real‐world rates of suicide‐specific mortality among adult patients (aged ≥ 18 years) with TRD, MDD+SI/SA, or MDD alone. The sampling frame comprised commercial and Medicare enrollees with medical and Part D coverage (MAPD) in the Optum Research Database (ORD) with evidence of at least two medical claims with a diagnosis of MDD on separate dates of service during the identification period (January 1, 2016, to June 30, 2019) (**Figure** **1**). Similar requirements have been used previously (Solberg, 2006). Study cohorts included patients who met the diagnostic criteria of TRD, MDD+SI/SA, and MDD alone during the identification period with at least a 6‐month history prior to the index diagnosis (defined in Section 2.2). The 6‐month observation before the index date was specified to ensure complete medical data. Diagnoses could appear in either the primary or the secondary position on the medical claim. The current study assessed the presence of SI/SA at baseline and in the last year of observation, defined as 365 days before the earliest of the following: patients’ disenrollment from the health plan, death, or September 30, 2019 (inclusive). The last year of observation was analyzed to standardize risk by capturing outcomes during a defined period of established disease. Data were linked with the National Death Index (NDI) to determine fact and cause of death.

### 2.2 Patient cohorts

Three patient cohorts were created: (1) TRD group (patients with treatment‐resistant MDD); (2) MDD+SI/SA group (patients with MDD and SI/SA); and (3) MDD alone group (patients with MDD who did not have TRD or SI/SA). Cohort identification algorithms were applied during the identification period. The TRD and MDD+SI/SA cohorts were not mutually exclusive. Patients with bipolar disorder/mania were excluded from all cohorts.

A healthcare database validated algorithm was used to define TRD (Cepeda et al., 2018). Following the date of established MDD, patients had to have at least three distinct antidepressants (at the ingredient level) within a 365‐day window or at least one antidepressant and at least one atypical antipsychotic within a 365‐day window during the identification period for inclusion in the TRD group. The TRD index date was the date of service associated with the latest qualifying pharmacy fill. Patients with a history of dementia or psychosis in the 6 months prior to TRD index date were excluded from this cohort. Established MDD for the TRD cohort was defined as two or more medical claims with a diagnosis of MDD on separate dates of service within a 365‐day window or an MDD‐related inpatient stay (an inpatient stay with a diagnosis of MDD, in any position, at any time during the inpatient stay). The earliest date of service associated with the second MDD diagnosis or discharge date for a qualifying inpatient stay was denoted as the date of established MDD (see **Table** **S1**).

For inclusion in the MDD+SI/SA group, patients had to have at least one medical claim with a diagnosis, in any position, of SI (ICD‐10‐Clinical Modification [ICD‐10‐CM] diagnosis code: R45.851) and/or SA (ICD‐10‐CM diagnosis codes: T‐36–T‐65, T71) following established MDD during the identification period. The SI/SA index date was the earliest date of service associated with a qualifying SI/SA claim. SI/SA was attributed to MDD if there was at least one medical claim with a diagnosis, in any position, of MDD within 365 days prior to SI/SA index date or at least one MDD‐related emergency room encounter or at least one MDD‐related inpatient stay at any time prior to SI/SA index date. Patients with a prior history of dementia, autism, or psychosis in the 6 months prior to SI/SA index date were excluded from this cohort.

Two algorithms that were created to identify SAs in claims databases and validated against medical‐chart review were used. The algorithms followed a modified version of Simon and colleagues’ algorithm, which determined self‐harm or a probable suicide attempt from ICD‐9 codes indicating intentional self‐harm or undetermined intent (Simon et al., 2018). The algorithms in the current study did not include injuries of uncertain intent, however, because Barak‐Corren and colleagues’ algorithm (Barak‐Corren et al., 2017) found these codes had low predictive values. The included codes were related to self‐inflicted injury and drug poisoning. Equivalent ICD‐10 codes were added to cover the recent data and concepts/codes for SI/thoughts (Cepeda et al., 2020). These algorithms reported positive predictive values ranging from 70% to 100%.

The MDD alone cohort comprised patients with at least two medical claims with a diagnosis of MDD, in any position, on separate dates of service during the identification period who did not meet TRD or SI/SA inclusion criteria.

### 2.3 Variables of interest

Variables of interest included patient sex; age (calculated as of the year of index date); comorbid conditions and psychotropic‐medication use during the 6‐month baseline period and 1 year prior to the end of the observation; and geographic region of the United States in which the patient was enrolled in a health plan. General comorbid conditions were defined using the Clinical Classifications Software managed by the Agency for Healthcare Research and Quality. This measure generated indicator variables for specific disease conditions based on ICD‐9 and ICD‐10 diagnosis codes. For SI and SA, indicator variables were created as well. SI and SA diagnoses were captured in any position at baseline and during the last year of observation period.

### 2.4 Data sources

The study was conducted using the ORD and the NDI. The ORD, which is Optum's proprietary administrative claims research database, is among the largest and most complete databases in the United States and is fully de‐identified, Health Insurance Portability and Accountability Act of 1996‐compliant, and comprises medical and pharmacy claims data on more than 67 million individuals. ORD's features include being eligibility‐controlled and displaying complete member information along with enrollment dates and plan‐design details. In addition, medical and pharmacy claims are available in the database that deliver insight into all healthcare services. In 2016, approximately 19% of the nation's commercially enrolled population, 17% of the Medicare Advantage population, and 23% of the Medicare Part D population were represented in the ORD.

Patients who met the inclusion criteria and were not active in the ORD beyond the end of the observation period were linked to the NDI, which is a centralized database of death record information on file in state vital statistics offices available to investigators for research purposes in public health and medical studies (Centers for Disease Control & Prevention, 2021a). Cause‐of‐death codes were obtained using the NDI Plus service, which was established in 1997 following negotiations with the states that permit the National Center for Health Statistics to release underlying cause‐of‐death codes and multiple cause‐of‐death codes. The NDI returns date of death and, for verifiable (or true) matches, all the ICD information for cause(s) of death listed on the death certificate. ICD‐10 codes used to define completed suicide in conjunction with the fact of death included codes for accidental poisoning, intentional self‐poisoning, intentional self‐harm, poisonings of undetermined intent, accidents of undetermined intent, and sequelae of intentional self‐harm, events of undetermined intent, and unspecified external cause (see **Table** **S2**). Additionally, a sensitivity analysis was conducted using two additional suicide definitions: a probable definition that excluded poisonings and accidents of undetermined intent and a strict definition that excluded accidental poisoning (see **Table** **S3**). NDI‐sourced fact and cause‐of‐death information (suicide or not) was deterministically linked to patient records.

### 2.5 Statistical analysis

Three cohorts were captured. Univariate and bivariate descriptive statistics for the sample disposition, descriptive statistics about patient characteristics of each cohort (age, sex, comorbidities, and medication history), and suicide‐specific mortality in each group were calculated.

#### 2.5.1 Calculation of time‐at‐risk

The mortality rates due to suicide were estimated as the number of observed suicides divided by person‐years‐at‐risk. Patients were considered at risk starting at the index date and contributed time‐at‐risk until the earliest of disenrollment from the health plan, end of the observation period, or death. Patients were censored if disenrollment from the health plan or the end of the observation period occurred prior to the date of death. Mortality rates were also stratified by age, sex, and the presence of recent SI/SA (defined as the last year of observation).

### 2.6 Protection of human subjects

The study was a secondary analysis of de‐identified data, which was reviewed by the New England Institutional Review Board (IRB) and determined to be exempt because it does not meet the definition of human subject research (NEIRB# 17‐1298974‐1).

## 3 RESULTS

### 3.1 Patients

**FIGURE 2:**
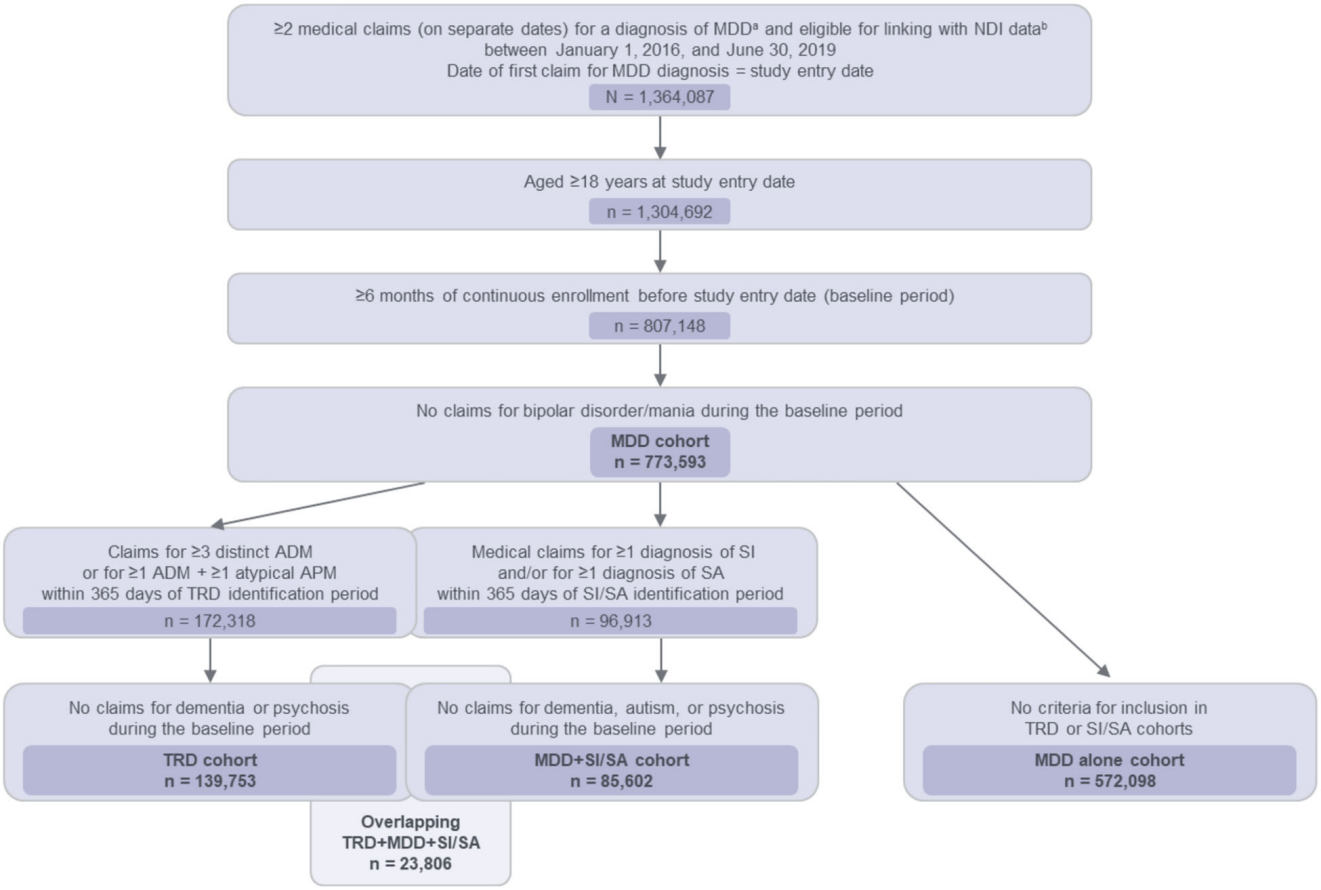
**Patient identification, attrition, and cohorts**. ^a^Diagnosis codes for MDD could appear in either the primary or secondary position. ^b^Fully insured on a commercial health plan or enrolled in a nonretiree Medicare Advantage with Part D coverage health plan. ADM, antidepressant medication; APM, antipsychotic medication; MDD, major depressive disorder; NDI, National Death Index; SA, suicide attempt; SI, suicidal ideation; TRD, treatment‐resistant depression.

A total of 807,148 patients had MDD and met the inclusion criteria. Patient attrition for the three cohorts of interest is described in **Figure** **2**. A total of 139,753 adult patients were identified for the TRD cohort; 85,602 for the MDD+SI/SA cohort; and 572,098 for the MDD alone cohort. A total of 23,806 patients were included in both the TRD and MDD+SI/SA cohorts. The 377,626 patients without enrollment beyond December 31, 2019, were searched for in the NDI. Patients with enrollment beyond the end of observation were assumed alive and censored accordingly.

Mean age ranged from 55 to 59 years in the three cohorts, and the majority of patients (64%−71%) were female (**Table** **1**). Anxiety disorders were present in 53.92% of patients with TRD, 44.11% of patients with MDD+SI/SA, and 21.72% of patients with MDD alone. Substance‐related disorders were present in 20.81%, 24.64%, and 9.03% of patients with TRD, MDD+SI/SA, and MDD alone, respectively. The percentage of patients with baseline exposure to antidepressants was 90.77%, 68.76%, and 55.11% in the TRD, MDD+SI/SA, and MDD alone cohorts, respectively; the percentages with adjunctive antipsychotic exposure at baseline were 17.11%, 5.91%, and 4.03%, respectively. The percentage of patients with anxiolytic or benzodiazepine exposure was lower in the MDD alone cohort than in the TRD and MDD+SI/SA cohorts (**Table** **1**).

**TABLE 1:** **Baseline demographics and characteristics for patients in the three cohorts** ^a^.

**Baseline demographic/characteristic**	**TRD** **(*n* = 139,753)**	**MDD + SI/SA** **(*n* = 85,602)**	**MDD alone** **(*n* = 572,098)**
**Age (continuous), years**			
Mean (SD)	55.14 (17.01)	57.91 (18.29)	58.74 (18.84)
Median (IQR)	57.00 (43.00−68.00)	61.00 (47.00−71.00)	62.00 (45.00−73.00)
**Age group, *n* (%)**			
18−44	36,820 (26.35)	19,254 (22.49)	141,731 (24.77)
45−64	55,294 (40.28)	29,284 (34.21)	170,907 (29.87)
65+	46,639 (33.37)	37,064 (43.30)	259,460 (45.35)
**Sex, *n* (%)**			
Female	98,724 (70.65)	55,075 (64.35)	385,065 (67.32)
Male	41,011 (29.35)	30,505 (35.65)	186,932 (32.68)
**Medical conditions, *n* (%)** ^a^			
Anxiety disorders	75,358 (53.92)	37,762 (44.11)	124,232 (21.72)
Attention deficit conduct and disruptive behavior disorders	8,517 (6.09)	3,094 (3.61)	15,080 (2.64)
Cerebrovascular disease	10,540 (7.54)	9,855 (11.51)	45,347 (7.93)
Dementia^b^	0 (0.00)	0 (0.00)	46,521 (8.13)
Epilepsy; convulsions	5,535 (3.96)	4,757 (5.56)	14,929 (2.61)
Headache, including migraine	23,625 (16.90)	14,291 (16.69)	49,264 (8.61)
Intracranial injury	1,730 (1.24)	1,335 (1.56)	5,303 (0.93)
Personality disorders	1,641 (1.17)	695 (0.81)	1,681 (0.29)
Psychosis^b^	0 (0.00)	0 (0.00)	14,399 (2.52)
Schizophrenia and other psychotic disorders	81 (0.06)	29 (0.03)	14,357 (2.51)
Substance‐related disorders	29,088 (20.81)	21,089 (24.64)	51,688 (9.03)
Suicide and intentional self‐inflicted injury	1,327 (0.95)	1,869 (2.18)	2,568 (0.45)
**Medications, *n* (%)**			
Antidepressant	126,858 (90.77)	58,858 (68.76)	315,269 (55.11)
Antipsychotic	23,910 (17.11)	5,058 (5.91)	23,032 (4.03)
Anxiolytic	69,613 (49.81)	35,001 (40.89)	133,348 (23.31)
Benzodiazepine	61,401 (43.94)	41,785 (37.13)	121,951 (21.32)

*Note*: Patients were required to have 6 months of data prior to the index date, not inclusive of the index date, to capture baseline characteristics and demographics.

^a^Clinical Classification Software for ICD‐9‐CM/ICD‐10‐CM. Agency for Healthcare Research and Quality, Rockville, MD. http://www.hcup‐us.ahrq.gov/toolssoftware/ccs/ccs.jsp; http://www.hcup‐us.ahrq.gov/toolssoftware/ccs10/ccs10.jsp.

^b^Not Agency for Healthcare Research and Quality classification.

ICD‐9‐CM, International Classification of Diseases‐9‐Clinical Modification; ICD‐10‐CM, International Classification of Diseases‐10‐Clinical Modification; IQR, interquartile range; MDD, major depressive disorder; SA, suicide attempt; SI, suicidal ideation; TRD, treatment‐resistant depression.

### 3.2 Suicide rates

Rates of suicide were 0.14 per 100 person‐years for patients with TRD, 0.27 per 100 person‐years for patients with MDD+SI/SA, and 0.04 per 100 person‐years for patients with MDD alone (**Table** **2**). Having SI or SA during the year prior to the end of observation (first of death, end of enrollment, or end of study period) markedly increased the risk of suicide by more than 10 times.

**TABLE 2:** Incidence rates of suicide overall and in patients with SI/SA during the last year of observation in the three cohorts^.^

	**TRD** **(*n* = 139,753)**			**MDD+SI/SA** **(*n* = 85,602)**			**MDD alone** **(*n* = 572,098)**		
**Suicide**	**Events**	**Person‐years**	**Rate** **per 100**	**Events**	**Person‐years**	**Rate** **per 100**	**Events**	**Person‐years**	**Rate** **per 100**
**Overall**	261	191,286	0.1364	289	108,377	0.2667	352	936,048	0.0376
**Patients with SI or SA during the last year of observation (death, censor)**	114	3,405	3.3480	254	8,936	2.8425	38	4,462	0.8516

*Note*: Suicide was defined using ICD‐10 codes for accidental poisoning (X40–X44), intentional self‐poisoning (X60–X69), intentional self‐harm (X70–X84), poisonings of undetermined intent (Y10–Y19), accidents of undetermined intent (Y20–Y34), sequelae of intentional self‐harm (Y87.0), sequelae of events of undetermined intent (Y87.2), and sequelae of unspecified external cause (Y89.9).

ICD‐10, International Classification of Diseases‐10; MDD, major depressive disorder; SA, suicide attempt; SI, suicidal ideation; TRD, treatment‐resistant depression.

Stratifying by age and sex found that rates of suicide were roughly three times higher in men than women across each of the three cohorts. Suicide was less frequent in patients aged 65 years and older, with rates roughly three to four times higher in those aged 18−44 and 45−64 years (**Table** **3**).

**TABLE 3:** Incidence rates of suicide among patients stratified by age and sex.

	**TRD** **(*n* = 139,753)**			**MDD + SI/SA** **(*n* = 85,602)**			**MDD alone** **(*n* = 572,098)**		
**Demographic**	**Events**	**Person‐years**	**Rate** **per 100**	**Events**	**Person‐years**	**Rate** **per 100**	**Events**	**Person‐years**	**Rate** **per 100**
**Age group**									
18−44	78	37,984	0.2054	91	19,822	0.4591	94	169,876	0.0553
45−64	136	75,617	0.1799	142	36,086	0.3935	157	266,494	0.0589
65+	47	77,685	0.0605	56	52,469	0.1067	101	499,677	0.0202
**Sex**									
Female	115	136,451	0.0843	114	71,747	0.1589	136	641,886	0.0212
Male	146	54,814	0.2664	175	36,611	0.4780	215	294,035	0.0731

MDD, major depressive disorder; SA, suicide attempt; SI, suicidal ideation; TRD, treatment‐resistant depression.

## 4 DISCUSSION

The current study using data from the ORD and the NDI found that the rates of death by suicide was 0.14, 0.27, and 0.04 per 100 person‐years for adult patients with TRD, MDD+SI/SA, and MDD alone, respectively. Suicide rates were highest for younger patients and men. Rates of suicide increased substantially with recent SI/SA.

MDD, especially when complicated by treatment resistance, is a common psychiatric disorder associated with high risk of suicide; approximately 15% of patients with MDD are at high risk for suicide, and 30% of patients with TRD are estimated to have at least one SA (Bachmann, 2018; Bergfeld et al., 2018; Orsolini et al., 2020; WHO, 2017). The current study found that rates of suicide were high in patients with TRD. Previous studies evaluating mortality and suicide risk in patients with MDD and TRD have shown greater rates of attempted suicide, all‐cause mortality, and suicide‐related mortality for patients with TRD (Brenner et al., 2021; Dold et al., 2018; Gronemann et al., 2021; Li et al., 2019; Nelsen & Dunner, 1995; Reutfors et al., 2018). Compared with patients with MDD, an increased suicide‐related mortality has been observed in patients with TRD even at mild levels of depression symptoms (Dold et al., 2018; Li et al., 2019). In a recent study, patients with TRD had a higher percentage of deaths due to traceable external causes (i.e., suicides and accidents) than patients with MDD (50% vs. 37%; Brenner et al., 2021). An age‐sex matched study demonstrated that patients with TRD were more likely to have made a SA than those with MDD (Nelsen & Dunner, 1995).

Rehospitalization for SI/SA within a year is common among patients with MDD previously hospitalized for SI/SA (Cepeda et al., 2020). Although SAs are up to 30 times more common than suicides, they are the most important predictors of repeated SAs and suicide (Bachmann, 2018; Ribeiro et al., 2016; WHO, 2021). In line with these previous reports, the current study found high suicide rates in patients with MDD+SI/SA, which increased substantially in patients with recent SI/SA (1 year before death). Other studies showed increased rates of suicide in patients with MDD+SI/SA, especially in those patients with recent SI/SA (Li et al., 2017; Reutfors et al., 2018, 2021; Simon et al., 2018; Whittier et al., 2016). Although recent SI and SA before death were observed and were confirmed as risk factors for suicide across cohorts in the current study, it is noteworthy that suicide rates in this study were 24 times higher (from 0.14 per 100 patient‐years to 3.35 per 100 patient‐years) in patients with TRD and recent SI/SA compared with the overall TRD cohort. This study examined groups of all patients with TRD and all patients with MDD+SI/SA, but not their mutually exclusive subgroups (e.g., patients with TRD with SI/SA and those without SI/SA). Exploring these sub‐cohorts and the interaction between treatment resistance and history of SI/SA within patients with MDD and the rates of suicide in each sub‐cohort could be a topic for future research.

Previous reports have highlighted the importance of including undetermined intent and accidental poisoning codes in suicide definitions because 90% and 80% of cases coded as undetermined and accidental drug deaths, respectively, are suicide (Rockett, 2020; Rockett, 2016). In our sensitivity analysis, suicides in the TRD cohort dropped from 261 with the original definition to 248 without undetermined intent codes. Of the 13 cases lost, 90% (12 cases) are expected to be suicides. With accidental poisonings also excluded, 118 additional cases were lost; we expect 80% (94 cases) of these to be suicides. This leaves 236 true suicides of the 261 originally identified suicides, a 10% overestimation (i.e., 90% positive predictive value, 100% sensitivity). With the strict definition, only 130 of 236 true suicides were captured, a 45% underestimation (i.e., 100% positive predictive value, 55% sensitivity). Of these options, we chose the method with the lesser bias.

It is well established that depression is strongly related to SI/SA, but limited knowledge exists regarding the patient characteristics that increase suicide risk among patients with MDD and TRD, possibly due to differences that arise between regions and countries with respect to age, sex, and other variables (Brådvik, 2018; Bachmann, 2018). The current study found higher suicide rates in male patients compared with female patients. This aligns with the recent medical literature reporting that completed suicides are up to three times more common in men than women, even though SAs are significantly more common in female patients (Bachmann, 2018; Brådvik, 2018; Handley et al., 2018; WHO, 2017, 2021; Finkelstein, 2015). However, these trends may be changing, as suicide rates increased by 55% in women from 2006 to 2018 compared with an overall increase of 28% during that entire reporting period of 1999−2018 (Hedegaard et al., 2020). Although it has been reported that suicides tend to occur more often in elderly than younger patients with depression (WHO, 2021; Bachmann, 2018; Hedegaard et al., 2020), the current study found the opposite, as rates of suicide in patients ≥65 years of age were three to four times lower than rates of suicide in patients aged 18–44 years. This supports a recent report that suicide rates among adults between 25 and 44 years of age have surpassed rates seen in older adults (aged ≥65 years) (Centers for Disease Control & Prevention, 2021b).

The current study used a robust healthcare database, the ORD, that included numerous patients. In addition, it allowed for linking to the NDI, the gold standard to study suicide rates, for fact and cause of death with the large population size. In terms of limitations, however, this study was restricted to enrollees of a commercial health plan or Medicare Advantage. The study was able to assess adults of all ages, and although patients 65 years of age or older are present, patient records required employment or Medicare Advantage coverage information to be included; therefore, the study excluded many of the elderly as well as those not eligible for employer‐based insurance.

It is possible that the rates of completed suicides could differ from the broader population among employed individuals and their dependents. Our chosen observation window was relatively short (2.5 years). Although the report of death is likely to be accurate, suicide as the cause of death was likely underreported (WHO, 2021). To mitigate the possibility of misclassification, a combination of suicides by any method and “accidents” of undetermined cause were considered death owing to suicide based on published data indicating that 80% of “accidental” drug intoxication deaths and 90% of undetermined drug intoxication deaths could be considered suicide (Rockett, 2020; Rockett, 2016). An additional limitation is that SI may be under‐captured in medical claims data, as reported previously (Pilon et al., 2022).

## 5 CONCLUSION

This descriptive study using the ORD and the NDI databases found that incidences of death due to suicide were 0.14, 0.27, and 0.04 per 100 person‐years for the TRD, MDD+SI/SA, and MDD alone cohorts, respectively. Suicide rates were substantially higher in patients with recent SI/SA. This study also found that suicide rates are higher in younger adults and were more prevalent in men than women. The high prevalence of suicidality (SI/SA) and suicide among patients with MDD and TRD seen in the current study suggests the need for a careful assessment and determination of suicide risk in this patient population.

## AUTHOR CONTRIBUTIONS

All authors and the funder of this study participated in study design. Due to the sensitive nature of the study, only Optum employees had access to and analyzed the full dataset. All authors reviewed and interpreted the final datasets for the MDD, TRD, and MDD+SI/SA cohorts, participated in the development and critical review of the manuscript, approved submission of the manuscript for publication, and are accountable for the accuracy and integrity of the work.

## CONFLICT OF INTEREST STATEMENT

DMK, CMC, JJ, DJF, and TD are employees of Janssen Research & Development, LLC. MSC and ED are former employees of Janssen Research & Development, LLC, and were employees during the study development, data analysis, and manuscript development. JCJ and CBP are employees of Optum. Janssen Research & Development, LLC, has an interest in depression and treatment‐resistant depression.

## DATA AVAILBILITY STATEMENT

The data that support the findings of this study are available from Optum. Restrictions apply to the availability of these data, which were used under license for this study. Data are available from the authors with the permission of Optum.

### PEER REVIEW

The peer review history for this article is available at https://publons.com/publon/10.1002/brb3.3171.

## Supporting information

Supp Information
