# Supplementary material for: Suicide‐specific mortality among patients with treatment‐resistant major depressive disorder, major depressive disorder with prior suicidal ideation or suicide attempts, or major depressive disorder alone
Source: Brain Behav. 2023 Jul 21;13(8):e3171. doi: 10.1002/brb3.3171 (PMC10454258; doi:10.1002/brb3.3171)
Supplement: Supplementary file 1 — Supp Information [file BRB3-13-e3171-s001.docx]

**SUPPLEMENTAL MATERIAL**

**Table S1. ICD-10 codes used to define major depressive disorder**

| **ICD code** | **Name** |
| --- | --- |
| F32.4 | Major depressive disorder, single episode, in partial remission |
| F33.42 | Major depressive disorder, recurrent, in full remission |
| F33.41 | Major depressive disorder, recurrent, in partial remission |
| F33.40 | Major depressive disorder, recurrent, in remission, unspecified |
| F32.5 | Major depressive disorder, single episode, in full remission |
| F32.89 | Other specified depressive episodes |
| F32.81 | Premenstrual dysphoric disorder |
| F34.1 | Dysthymic disorder |
| F33.9 | Major depressive disorder, recurrent, unspecified |
| F33.8 | Other recurrent depressive disorders |
| F33.3 | Major depressive disorder, recurrent, severe with psychotic symptoms |
| F33.2 | Major depressive disorder, recurrent severe without psychotic features |
| F33.1 | Major depressive disorder, recurrent, moderate |
| F33.0 | Major depressive disorder, recurrent, mild |
| F32.9 | Major depressive disorder, single episode, unspecified |
| F32.8 | Other depressive episodes |
| F32.3 | Major depressive disorder, single episode, severe with psychotic features |
| F32.2 | Major depressive disorder, single episode, severe without psychotic features |
| F32.1 | Major depressive disorder, single episode, moderate |
| F32 | Major depressive disorder, single episode |
| F32.0 | Major depressive disorder, single episode, mild |
| F53.0 | Postpartum depression |
| F33 | Major depressive disorder, recurrent |
| F33.4 | Major depressive disorder, recurrent, in remission |

ICD, International Classification of Diseases.

**Table S2. ICD-10 codes used to define completed suicide in conjunction with fact of death**

| **ICD code** | **Name** |
| --- | --- |

| X40 | Accidental poisoning by and exposure to nonopioid analgesics, antipyretics and antirheumatics |
| --- | --- |
| X41 | Accidental poisoning by and exposure to antiepileptic, sedative-hypnotic, antiparkinsonism and psychotropic drugs, not elsewhere classified |
| X42 | Accidental poisoning by and exposure to narcotics and psychodysleptics [hallucinogens], not elsewhere classified |
| X43 | Accidental poisoning by and exposure to other drugs acting on the autonomic nervous system |
| X44 | Accidental poisoning by and exposure to other and unspecified drugs, medicaments and biological substances |
| X60 | Intentional self-poisoning by and exposure to nonopioid analgesics, antipyretics and antirheumatics |
| X61 | Intentional self-poisoning by and exposure to antiepileptic, sedative-hypnotic, antiparkinsonism and psychotropic drugs, not elsewhere classified |
| X62 | Intentional self-poisoning by and exposure to narcotics and psychodysleptics [hallucinogens], not elsewhere classified |
| X63 | Intentional self-poisoning by and exposure to other drugs acting on the autonomic nervous system |
| X64 | Intentional self-poisoning by and exposure to other and unspecified drugs, medicaments and biological substances |
| X65 | Intentional self-poisoning by and exposure to alcohol |
| X66 | Intentional self-poisoning by and exposure to organic solvents and halogenated hydrocarbons and their vapours |
| X67 | Intentional self-poisoning by and exposure to other gases and vapours |
| X68 | Intentional self-poisoning by and exposure to pesticides |
| X69 | Intentional self-poisoning by and exposure to other and unspecified chemicals and noxious substances |
| X70 | Intentional self-harm by hanging, strangulation and suffocation |
| X71 | Intentional self-harm by drowning and submersion |
| X72 | Intentional self-harm by handgun discharge |
| X73 | Intentional self-harm by rifle, shotgun and larger firearm discharge |
| X74 | Intentional self-harm by other and unspecified firearm discharge |
| X75 | Intentional self-harm by explosive material |
| X76 | Intentional self-harm by smoke, fire and flames |
| X77 | Intentional self-harm by steam, hot vapours and hot objects |
| X78 | Intentional self-harm by sharp object |
| X79 | Intentional self-harm by blunt object |
| X80 | Intentional self-harm by jumping from a high place |
| X81 | Intentional self-harm by jumping or lying before moving object |
| X82 | Intentional self-harm by crashing of motor vehicle |
| X83 | Intentional self-harm by other specified means |
| X84 | Intentional self-harm by unspecified means |
| Y10 | Poisoning by and exposure to nonopioid analgesics, antipyretics and antirheumatics, undetermined intent |
| Y11 | Poisoning by and exposure to antiepileptic, sedative-hypnotic, antiparkinsonism and psychotropic drugs, not elsewhere classified, undetermined intent |
| Y12 | Poisoning by and exposure to narcotics and psychodysleptics [hallucinogens], not elsewhere classified, undetermined intent |
| Y13 | Poisoning by and exposure to other drugs acting on the autonomic nervous system, undetermined intent |
| Y14 | Poisoning by and exposure to other and unspecified drugs, medicaments and biological substances, undetermined intent |
| Y15 | Poisoning by and exposure to alcohol, undetermined intent |
| Y16 | Poisoning by and exposure to organic solvents and halogenated hydrocarbons and their vapours, undetermined intent |
| Y17 | Poisoning by and exposure to other gases and vapours, undetermined intent |
| Y18 | Poisoning by and exposure to pesticides, undetermined intent |
| Y19 | Poisoning by and exposure to other and unspecified chemicals and noxious substances, undetermined intent |
| Y20 | Hanging, strangulation and suffocation, undetermined intent |
| Y21 | Drowning and submersion, undetermined intent |
| Y22 | Handgun discharge, undetermined intent |
| Y23 | Rifle, shotgun and larger firearm discharge, undetermined intent |
| Y24 | Other and unspecified firearm discharge, undetermined intent |
| Y25 | Contact with explosive material, undetermined intent |
| Y26 | Exposure to smoke, fire and flames, undetermined intent |
| Y27 | Contact with steam, hot vapours and hot objects, undetermined intent |
| Y28 | Contact with sharp object, undetermined intent |
| Y29 | Contact with blunt object, undetermined intent |
| Y30 | Falling, jumping or pushed from a high place, undetermined intent |
| Y31 | Falling, lying or running before or into moving object, undetermined intent |
| Y32 | Crashing of motor vehicle, undetermined intent |
| Y33 | Other specified events, undetermined intent |
| Y34 | Unspecified event, undetermined intent |
| Y87.0 | Sequelae of intentional self-harm |
| Y87.2 | Sequelae of events of undetermined intent |
| Y89.9 | Sequelae of unspecified external cause |

ICD, International Classification of Diseases.

**Table S3. Sensitivity analysis of three definitions of suicide overall and in patients with SI/SA during the last year of observation in the three cohorts**

| **Suicide** | **TRD** **(n=139,753)** | | | **MDD+SI/SA** **(n=85,602)** | | | **MDD alone** **(n=572,098)** | | |
| --- | --- | --- | --- | --- | --- | --- | --- | --- | --- |
|  | **Events** | **Person-time** | **Rate** **per 100** | **Events** | **Person-years** | **Rate** **per 100** | **Events** | **Person-years** | **Rate** **per 100** |
| **Original definition^a^** | | | | | | | | | |
| **Overall** | 261 | 191,286 | 0.1364 | 289 | 108,377 | 0.2667 | 352 | 936,048 | 0.0376 |
| **Patients with SI or SA during the last year of observation (death, censor)** | 114 | 3405 | 3.3480 | 254 | 8936 | 2.8425 | 38 | 4462 | 0.8516 |
| **Probable definition^b^** | | | | | | | | | |
| **Overall** | 248 | 191,286 | 0.1296 | 276 | 108,377 | 0.2547 | 342 | 936,048 | 0.0365 |
| **Patients with SI or SA during the last year of observation (death, censor)** | 108 | 3405 | 3.1718 | 245 | 8936 | 2.7418 | 37 | 4462 | 0.8292 |
| **Strict definition**^c^ | | | | | | | | | |
| **Overall** | 130 | 191,286 | 0.0680 | 142 | 108,377 | 0.1310 | 198 | 936,048 | 0.0212 |
| **Patients with SI or SA during the last year of observation (death, censor)** | 51 | 3405 | 1.4978 | 123 | 8936 | 1.3765 | 18 | 4462 | 0.4034 |

ICD, International Classification of Diseases; MDD, major depressive disorder; SA, suicidal attempt; SI, suicidal ideation; TRD, treatment-resistant depression.

^a^The original definition defined suicide using ICD-10 codes for accidental poisoning (X40–X44), intentional self-poisoning (X60–X69), intentional self-harm (X70–X84), poisonings of undetermined intent (Y10–Y19), accidents of undetermined intent (Y20–Y34), sequelae of intentional self-harm (Y87.0), sequelae of events of undetermined intent (Y87.2), and sequelae of unspecified external cause (Y89.9).
^b^The probable definition defined suicide using ICD-10 codes for accidental poisoning (X40–X44), intentional self-poisoning (X60–X69), and intentional self-harm (X70–X84).
^c^The strict definition defined suicide using ICD-10 codes for intentional self-poisoning (X60–X69) and intentional self-harm (X70–X84).
